# Impact of four kVp combinations available in a dual‐source CT on the spectral performance of abdominal imaging: A task‐based image quality assessment on phantom data

**DOI:** 10.1002/acm2.13369

**Published:** 2021-07-26

**Authors:** Joël Greffier, Julien Frandon, Alexandre Sadate, Philippe Akessoul, Asmaa Belaouni, Jean‐Paul Beregi, Djamel Dabli

**Affiliations:** ^1^ Department of medical imaging Nîmes Medical Imaging Group CHU Nimes Univ Montpellier Nimes France

**Keywords:** dual‐energy imaging, dual‐source CT scan, spectral performance, task‐based image quality assessment, virtual monoenergetic images

## Abstract

**Purpose:**

To compare the spectral performance of four combinations of kVp available in a third generation dual‐source CT (DSCT) on abdominal imaging.

**Methods:**

An image‐quality phantom was scanned with a DSCT using four kVp pairs (tube “A” voltage/tube “B” voltage): 100/Sn150 kVp, 90/Sn150 kVp, 80/Sn150 kVp, and 70/Sn150 kVp, classic parameters and dose level for abdomen examination (CTDI_vol_: 11 mGy). The noise power spectrum (NPS) and the task‐based transfer function (TTF) of two inserts were computed on virtual monochromatic images (VMIs) at 40/50/60/70 keV and for mixed, low‐, and high‐kVp images. Detectability index (d’) was computed on VMIs and mixed images to model the detection task of liver metastasis (LM) and hepatocellular carcinoma (HCC). Iodine quantification accuracy was assessed using the Root Mean Square Deviation (RMSD_iodine_) and the iodine bias (IB).

**Results:**

Noise magnitude decreased by −55%± 0% between 40 and 70 keV for all kVp pairs. Compared to 70/Sn150 kVp, noise magnitude was increased by 9% ± 0% with 80/Sn150 kVp, by 16% ± 1% with 90/Sn150 kVp and by 24%± 1% with 100/Sn150 kVp. The average NPS spatial frequency (f_av_) shifted toward higher frequencies as energy level increased for all kVp pairs. Lowest f_av_ values were found for 70/Sn150 kVp and highest for 100/Sn150 kVp. The value of TTF at 50% (f_50_) shifted toward lower frequencies with increasing energy level. The highest f_50_ values occurred for 100/Sn150 kVp and the lowest for 80/Sn150 kVp. For both lesions, d’ was highest for 70/Sn150 kVp and lowest for 100/Sn150 kVp. Compared to 70/Sn150 kVp, d’ decreased by −6% ± 3% with 80/Sn150 kVp, by −11% ± 2% with 90/Sn150 kVp and by −13%± 2% with 100/Sn150 kVp. For all acquisitions, the RSMD_iodine_ and IB were the lowest for 100/Sn150 kVp (0.29 ± 0.10 mg/ml and 0.88 ± 0.30 mg/ml, respectively) and increased when the tube “A” voltage decreased (2.34 ± 0.29 mg/ml for 70/Sn150 kVp and 7.42 ± 0.51 mg/ml respectively).

**Conclusion:**

70/Sn150 kVp presented the lowest image noise and highest detectability in VMIs of two small focal liver lesions. 100/Sn150 kVp presented the lowest image noise on mixed images and highest accuracy of iodine quantification in iodine images.

## 1 INTRODUCTION

Since its launch, dual‐energy CT (DECT) imaging has played an important role in certain clinical applications, such as abdominal imaging.^1^, ^2^, ^3^, ^4^, ^5^ This technique is based on the attenuation differentiation of materials exposed simultaneously to low‐ and high‐energy X‐ray beams.^6^ Obtaining two measurement points for each volume measurement makes it possible to separately calculate the photon interactions by the photoelectric effect and by the Compton effect as a function of energy and allows material discrimination and quantification (e.g. iodine concentration).

Several DECT imaging techniques are currently commercially available,^7^, ^8^ including dual source CT‐scan (DSCT) using two pairs of X‐ray tubes and detectors mounted orthogonally (90° or 95° depending on the generation). Each tube is set to a different kVp value, where tube “A” delivers a low‐kVp beam and tube “B” delivers a high‐kVp beam, and the kVp pair is noted by low‐kVp/high‐kVp. The projection data acquired and reconstructed from each tube‐detector pair are used for material decomposition analyses. In the first generation of DSCT, one kVp pair was available (80/140 kVp). Another pair at 100/140 kVp was added in the same generation to improve image quality for obese patients due to tube power limitations at low‐kVp.^9^ In second generation DSCT, a tin filter was introduced in the X‐ray tube delivering the high‐kVp (tube “B”; 80/Sn140 kVp), improving the material discrimination via increased spectral separation.^10^ In the third generation, five pairs of kVp are available with or without the tin filter: 100/Sn150 kVp, 90/Sn150 kVp, 80/Sn150 kVp, 70/Sn150 kVp, and 80/140 kVp.

Different sets of images are generated in this third generation DSCT platform to allow spectral analyses and clinical interpretations. First, radiologists have access to the low‐kVp and high‐kVp images produced by each of the two X‐ray tubes. Then, mixed images are created from the low‐ and high‐kVp images to resemble the conventional 120 kVp image of single‐energy CT^6^ with higher photon statistics than the low‐ and high‐kVp images. Finally, spectral images are obtained from the basis material images (photoelectric and Compton) generated by the spectral decomposition in the image domain (“post‐reconstruction processing”) for DSCT. Virtual monoenergetic images (VMIs) are calculated as a linear combination of the basis material images with energy‐dependent coefficients, which approximate the energy dependence of the photoelectric interaction and the energy dependence of the total cross‐section for Compton scattering respectively.^11^, ^12^, ^13^ This type of images are useful for enhancing iodine contrast on abdominal lesions (such as focal liver lesions) at low monoenergetic levels (ranging from 40 to 60 keV) and reducing metallic artifacts at high monoenergetic levels (ranging from 140 to 190 keV). In addition, virtual contrast images or iodine maps are generated to enhance the visualization of the iodine contrast distribution. In clinical practice, radiologists preferentially use the mixed images and the VMIs at low‐keV to detect and characterize abdominal lesions such as renal or liver lesions.^1^, ^2^, ^3^, ^4^, ^5^

Several studies have evaluated and compared the spectral performances of different DECT platforms,^7^, ^14^, ^15^, ^16^, ^17^, ^18^, ^19^ but few studies have assessed the impact of the kVp pairs available in DSCT systems for spectral acquisition on spectral performance and image quality. Krauss et al.^9^ investigated the impact of the five pairs of kVp available in second and third generation DSCT on Filtered Back Projection images. They assessed the spectral separation of iodine material and the noise level of the combined and virtual non‐contrast images using different phantom sizes. However, this study did not assess the effect on image quality of mixed and separated low/high‐kVp images or of VMIs for the lowest energy levels.

The purpose of the present study was to assess the impact of four pairs of kVp (using a tin filter for high‐kVp tube “B”) available in a third generation DSCT on the spectral performance and image quality. Task‐based image quality metrics were used to assess the noise texture, noise magnitude, spatial resolution, and detectability of small focal liver lesions of mixed and separated low‐ and high‐kVp and VMIs at low‐keV. In addition, the accuracy of the iodine concentration was assessed for iodine images.

## 2 MATERIALS AND METHODS

### 2.1 Techniques for calculating mixed images

A mixed image is a weighted average of the original CT image. The CT values of a mixed image depend on three values: the CT value of the low energy image, the CT value of the high energy image and the dual energy composition. This latter is the low energy fraction of the image and it is determined by the DE composition parameter of the scan protocol. In this study, the DE composition used as default was 0.8.

The CT values of a mixed image are computed as follows:

$$x=w\times x_{\mathrm{low}}+\left(1‐w\right)\times x_{\mathrm{high}}$$

where x: CT value (HU) in image; w: the DE composition; $x_{\mathrm{low}}$ and $x_{\mathrm{high}}$: CT value in low and high energy images respectively.

VMIs are calculated as a linear combination of the basis material images with energy‐dependent coefficients which approximate the energy dependence of the photoelectric interaction and the energy dependence of the total cross‐section for Compton scattering respectively.

### 2.2 Phantoms used

**FIGURE 1:**
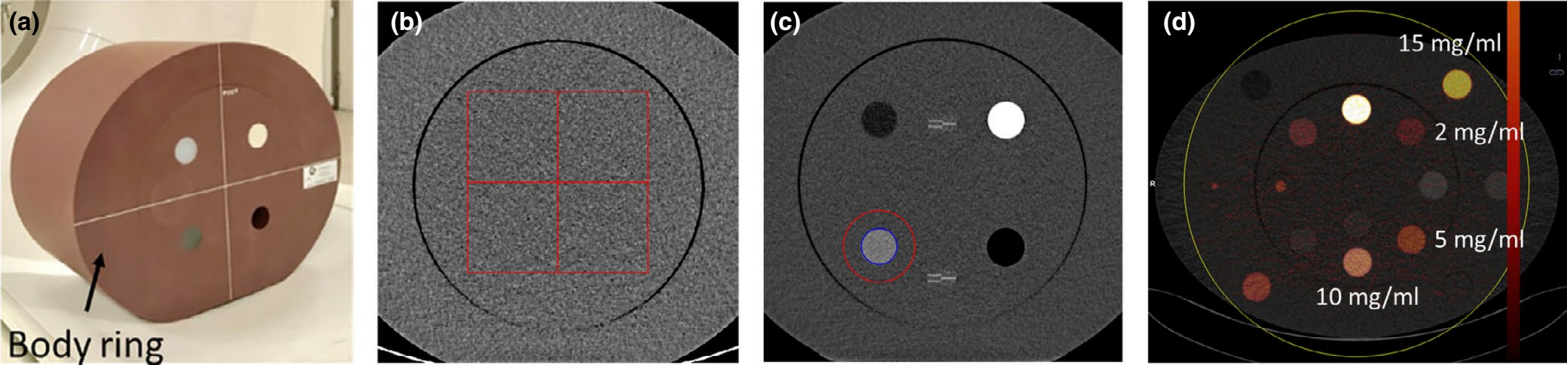
a. ACR phantoms placed inside a body ring; b. Four regions of interest (ROIs) of approximately 128x128 pixels used for the noise power spectrum (NPS) assessment; c. ROI used to compute the task‐based transfer function (TTF) placed on the acrylic insert; d. ROI used to measure the accuracy of the iodine concentration and placed on the 2, 5, 10 and 15 mg/ml iodine inserts

A 20‐cm diameter ACR QA phantom (Gammex 464, Middleton, WI) was placed inside a body ring (0 HU; Solid Water^®^; Gammex 464, Middleton, WI). The total size of the phantom used was 26.4 cm × 33 cm × 20 cm (Figure 1a) and was used to measure the noise power spectrum (NPS; Figure 1b) and the task‐based transfer function (TTF; Figure 1c). The phantom was placed inside its body ring to most closely simulate the morphology of patients undergoing an abdomen CT examination.

A 20‐cm diameter Multi‐Energy CT phantom was placed inside an elliptical ring (Sun Nuclear, Middleton, WI). The total size of the phantom used was 30 cm × 40 cm × 15 cm and was used to assess the accuracy of iodine concentration. This phantom was composed of removable inserts (diameter of 2.85 cm) placed into a water equivalent as background material. The inserts were placed in the same positions for each acquisition (Figure 1d).

### 2.3 Acquisition and reconstruction parameters

Acquisitions were performed with a third generation DSCT (Somatom Force, Siemens Healthineers, Forcheim, Germany). The acquisition parameters of classic abdomen examinations were used: a rotation time of 0.5 s/rot, pitch factor of 0.6 and beam collimation of 128 × 0.6 mm. Four pairs of low‐kVp in tube “A” and high‐kVp in tube “B” with an available tin filter were used: 100/Sn150 kVp, 90/Sn150 kVp, 80/Sn150 kVp, and 70/Sn150 kVp.

For each kVp pair, the reference quality tube current (mAs) can be adjusted for tube “A” but the mAs for tube “B” was automatically computed by the system. The tube current modulation system was activated for each acquisition to take into account the elliptical shape of each phantom. For each pair of kVp, the mAs of tube “A” was adjusted to obtain a CTDI_vol_ close to 11 mGy, which corresponds to the guide value of the French Diagnostic Reference Level (Table 1).

**TABLE 1:** Tube current for tube “A” and tube “B” voltages and mAs ratio between mAs used for both tubes before and after tube current modulation and volume CT dose index (CTDI_vol_) used for the four pairs of kVp

Tube voltage (Tube “A”/Tube “B”; kVp)		100/Sn150	90/Sn150	80/Sn150	70/Sn150
Tube current (mAs)	*Reference mAs*	275/138	319/199	446/223	800/200
	*Effective mAs*	183/99	211/143	295/160	525/143
mAs ratio	*Reference mAs*	1.99	1.60	2.00	4.00
	*Effective mAs*	1.85	1.48	1.84	3.67
Measured CTDI_vol_ (mGy)		10.71 ± 0.04	10.59 ± 0.02	10.64 ± 0.04	10.38 ± 0.03

Values of measured CTDI_vol_ are expressed as means ± standard deviations for the five acquisitions performed for each kVp pair.

Each phantom was centered on the CT scan isocentre and was scanned five times with the same acquisition and reconstruction parameters for each kVp pair.

Raw data were reconstructed with level 3 (A3) of advanced modeled iterative reconstruction (ADMIRE) using the standard soft tissue reconstruction kernel (Br40) usually used for abdomen exploration. Images were reconstructed with slice thickness of 1 mm (1‐mm increment) and a reconstruction field of view of 250 mm in the x‐y direction for the ACR phantom and 420 mm for the Multi‐energy phantom. These reconstruction parameters are used in clinical practice for abdomen CT.

The reconstructed images for the low‐ and high‐kVp were sent to Syngo.via software, which automatically generated a mixed image from the low‐ and high‐energy images. For each pair of kVp, virtual monochromatic images (VMIs) at 40, 50, 60, and 70 keV were generated using the “Monoenergetic +” application. Iodine concentration images were also computed using the “Virtual Unenhanced” application. Information about the "Monoenergetic +" and the "Virtual unenhanced" applications available in Syngo. Via software are detailed in Grant et al.^13^ and Johnson et al.^20^ respectively.

### 2.4 Task‐based image quality assessment

Task‐based image quality assessment was carried out using in‐house software developed by a working group of the French Society of Medical Physicists. This software was used to assess the noise texture and magnitude using the NPS and the spatial resolution using the TTF^21^, ^22^ with a similar methodology than that used by the imQuest software.^20^ The detectability index (d’) was used to model the detection of small focal lesions. For each of the five CT acquisitions performed, all metrics were computed for mixed and separated low‐ and high‐kVp images and VMIs at 40/50/60/70 keV.

#### 2.4.1 Noise power spectrum

**FIGURE 2:**
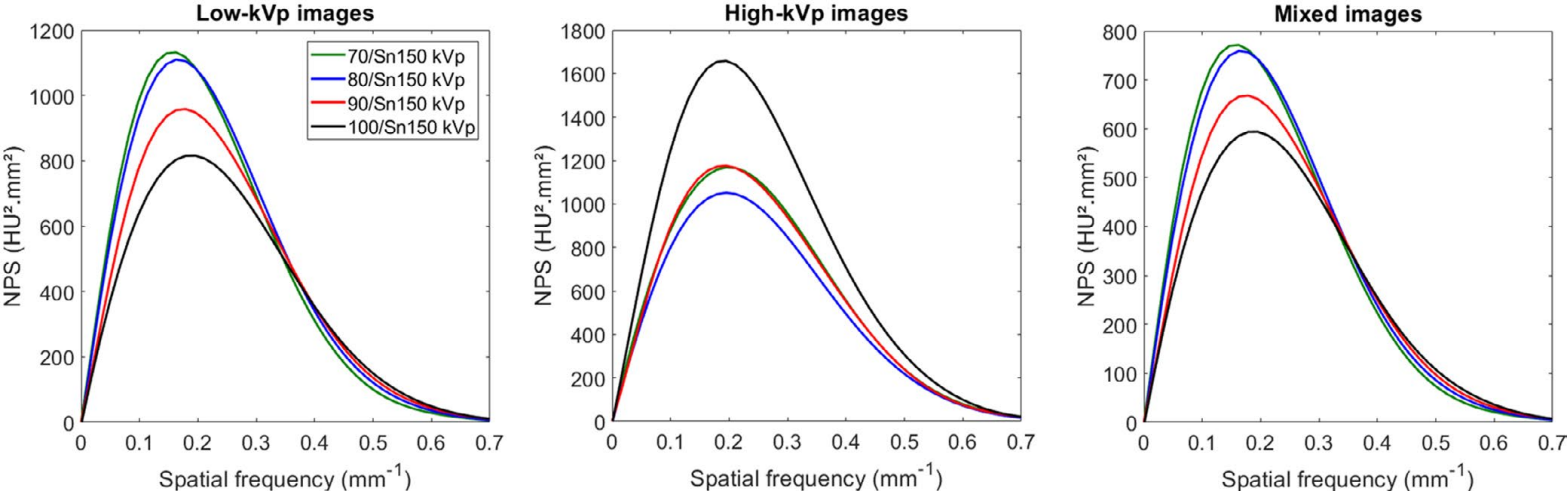
Noise power spectrum (NPS) curves obtained for all pairs of kVp for low‐kVp, high‐kVp, and mixed images. (Note that NPS curves for 70/Sn150 kVp and 90/Sn150 kVp [red and green colored curves] overlap for high‐kVp images)

The NPS was computed by placing four square regions of interest (ROIs) in the uniform section (module 3) of the ACR phantom (Figure 2) with the following equation:

$$NPS_{2D}\left(f_{x},f_{y}\right)=\frac{\Delta_{x}\Delta_{y}}{L_{x}L_{y}}\frac{1}{N_{\mathrm{ROI}}}\sum_{i=1}^{N_{\mathrm{ROI}}}{\left|FT_{2D}\left\{ROI_{i}\left(x,y\right)‐\bar{ROI_{i}}\right\}\right|}^{2}$$

where *Δ_x_* and *Δ_y_* are the pixel sizes in the x‐ and y‐directions, respectively, *L_x_* and *L_y_* are the ROI sizes in pixels along the x‐ and y‐axes, respectively, *N_ROI_* is the number of ROIs, *FT* is the Fourier transform and $\bar{ROI_{i}}$ is the mean pixel value measured from *ROI_i_(x*, *y)* allowing by subtraction the extraction of the noise from the pixel values of the *ROI_i_(x*, *y)*.^23^ This extraction technique, detrending, is used to remove the low‐frequency spike on the NPS curves.^23^, ^24^ The combined NPS was computed for a total of 80 ROIs (N_ROI_) of approximately 128 × 128 pixels (L_x_ and L_y_) each from 20 consecutive axial slices. The NPS curves obtained were fitted using the 11^th^‐order polynomial to smooth them.

The square root of the area under the NPS curve was used to quantify the noise magnitude and the average spatial frequency of the NPS curve (f_av_) for the noise texture.

#### 2.4.2 Task‐based transfer function

The TTF was assessed using acrylic insert available in module 1 of the ACR phantom from 10 consecutive axial slices according to the methodology previously reported^25^ and used.^21^, ^22^, ^26^, ^27^, ^28^ A circular ROI was placed around the insert, and a circular‐edge technique was employed to measure the edge spread function (ESF) by plotting the HU value of each pixel as a function of the distance to the center of the insert. The ESF was computed for each of the 10 axial slices and its median was computed. The line spread function (LSF) was then obtained by derivation of the median ESF. The TTF was computed from the normalized Fourier transformation of the LSF. Detailed process of the TTF calculation are depicted in the Supplementary Material file.

#### 2.4.3 Detectability index

A detectability index (d’) was computed to assess the detection of liver metastasis (LM) and hepatocellular carcinoma (HCC) on VMIs as function of energy levels and on mixed images. d’ combines the noise (NPS) and resolution (TTF) properties of the images with a predefined function, noted W, representative^22^ of a clinical imaging task to estimate how well a human observer would perform the considered task.

This index was based on a Non‐PreWhitening matched filter with Eye filter (NPWE) model observer as^21^, ^22^, ^25^:

$$d_{\mathrm{NPWE}}^{2}=\frac{{\left[\iint {\left|W\left(\text{u,v}\right)\right|}^{2}\cdot TTF{\left(\text{u,v}\right)}^{2}\cdot E{\left(\text{u,v}\right)}^{2}dudv\right]}^{2}}{\iint {\left|W\left(\text{u,v}\right)\right|}^{2}\cdot TTF{\left(\text{u,v}\right)}^{2}\cdot \text{NPS}{\left(\text{u,v}\right)}^{2}\cdot E{\left(\text{u,v}\right)}^{4}dudv}$$

where *u* and *v* are the spatial frequencies in the x‐ and y‐directions, *E* the eye filter that models the human visual system sensitivity to different spatial frequencies,^21^, ^29^ and *W(u*,*v)* the task function defined as:

$$W=\left|F\left\{h_{1}\left(x,y\right)‐h_{2}\left(x,y\right)\right\}\right|$$

where $h_{1}\left(x,y\right)$ and $h_{2}\left(x,y\right)$ correspond to the object present and the object absent hypotheses respectively.^23^, ^30^

The eye filter (E) was modeled according to the visual response function.^31^

The LM and HCC task functions were assumed to represent a circular signal with a diameter of 10 mm. To account for lesion enhancement variation as a function of keV, the contrast between each clinical task and the liver parenchyma was defined according to the curves of HU variations measured on patients and published by Wang et al. for LM, HCC, and the liver parenchyma^32^ (Table 2).

**TABLE 2:** Contrast between the hypodense liver metastasis or the hypodense hepatocellular carcinoma and the surrounding liver parenchyma as function of keV used to compute the detectability indexes. These values were extrapolated to the curves of HU variations measured on patients and published by Wang et al.^32^

Energy	Liver metastasis	Hepatocellular carcinoma
40 keV	−112 HU	−39 HU
50 keV	−78 HU	−30 HU
60 keV	−61 HU	−23 HU
70 keV	−50 HU	−17 HU
80 keV	−43 HU	−16 HU

Of note, as the lesions are hypodense, their HU values were lower than the surrounding liver parenchyma and hence the resulting contrast is negative.

The interpretation conditions used to obtain d’ included a 1.5 zoom factor, a viewing distance of 450 mm and a 500 mm field of view to refer to the visualization screen.

### 2.5 Iodine concentration

For each of the five CT acquisitions performed, the iodine concentration was measured in the iodine concentration images by placing a ROI (diameter of 2 cm) in the center of the four inserts at four iodine concentrations, 2, 5, 10 and 15 mg/ml (Figure 1d).

The accuracy of the iodine concentration was obtained using the root‐mean‐square deviation (RMSD_iodine_) between the measured and theoretical iodine concentration values using the following formula:

$$RMSD_{iodine}=\sqrt{\frac{\sum_{i=1}^{K}{\left(c_{\mathrm{theoretical}}^{i}‐c_{\mathrm{measured}}^{i}\right)}^{2}}{K}},\;\;c^{1,\ldots ,K}=2,5,10,15mg/ml.$$

The iodine bias (IB) was used to characterize each kVp pair's overall deviation from the nominal concentration^7^ and was computed, as follows:

$IB=\sum_{i=2,5,10,15}\left({\left[I\right]}_{i}‐i\right)$,

where i is the nominal iodine concentration and ${\left[I\right]}_{i}$is the iodine concentration measured for a given insert.

### 2.6 Dosimetry

For all kVp pairs, the CTDI_vol_ were measured using the 32‐cm diameter reference phantom and an ionization chamber. The ionization chamber was placed in the center of the phantom and then in each of the four cardinal peripheral positions. Five acquisitions were performed for each ionization chamber position and the CTDI_vol_ were computed.

## 3 RESULTS

### 3.1 Mixed and separated low‐ and high‐kVp images

#### 3.1.1 Tube current (mAs) and CTDI_vol_

The mAs of the tube “A” increased as tube voltage decreased (Table 1). For each kVp pair, the mAs used in tube “A” was 1.5 times higher than that in tube “B” for 90/Sn150 kV, 1.85 times for 100/Sn150 kV and 80/Sn150 kVp, and 3.7 times for 70/Sn150 kVp.

For all pairs of kVp, the measured CTDI_vol_ ranged from 10.38 ± 0.03 mGy to 10.71 ± 0.04 mGy. The ratio between the mean CTDI measured at the periphery and the center of the dosimetric phantom decreased when the tube “A” voltage increased (1.90 for 100/Sn150 kVp and 2.04 for 70/Sn100 kVp).

### 3.2 Noise power spectrum (NPS)

The NPS curves obtained for mixed, low‐ and high‐kVp images of each pair of kVp are shown in Figure 2. The lowest NPS peak values for low‐kVp and mixed images and the highest NPS peak value for high‐kVp images were found with 100/Sn150 kVp.

**FIGURE 3:**
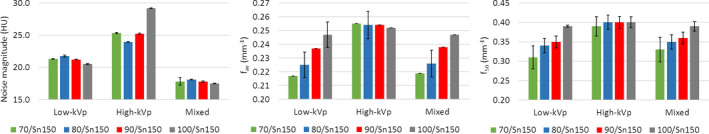
Mean values of noise magnitude, average NPS spatial frequency (f_av_), and TTF at 50% (f_50_) of acrylic insert and their respective error bars obtained for all pairs of kVp for low‐kVp, high‐kVp, and mixed images

For each pair of kVp, the noise magnitude was lower for mixed images than for low‐ and high‐kVp images, and was lower for low‐kVp images than for high‐kVp images (Figure 3). Similar values of noise magnitude were found on low‐kVp images (21.2 ± 0.5 HU) and on mixed images (17.8 ± 0.4 HU) as function of kVp pairs. For high‐kVp images, the highest noise magnitude was found with 100/Sn150 kVp.

For low‐kVp and mixed images, the average NPS spatial frequency (f_av_) shifted toward lower frequencies when the tube “A” voltage decreased (0.247 ± 0.002 mm^−1^ for 100/Sn150 kVp and 0.218 ± 0.004 mm^−1^ for 70/Sn150 kVp; Figure 3). For high‐kVp images, similar values of f_av_ were found for all pairs of kVp and all acquisitions (0.254 ± 0.001 mm^−1^).

### 3.3 Task‐based transfer function

For low‐kVp and mixed images, the highest values of TTF at 50% (f_50_) were obtained for 100/Sn150 kVp and decreased when the tube “A” voltage decreased (Figure 3). For high‐kVp, the f_50_ values were similar across all pairs of kVp and all acquisitions (0.394 ± 0.025 mm^−1^).

#### 3.3.1 Monochromatic images

### 3.4 Noise power spectrum

**FIGURE 4:**
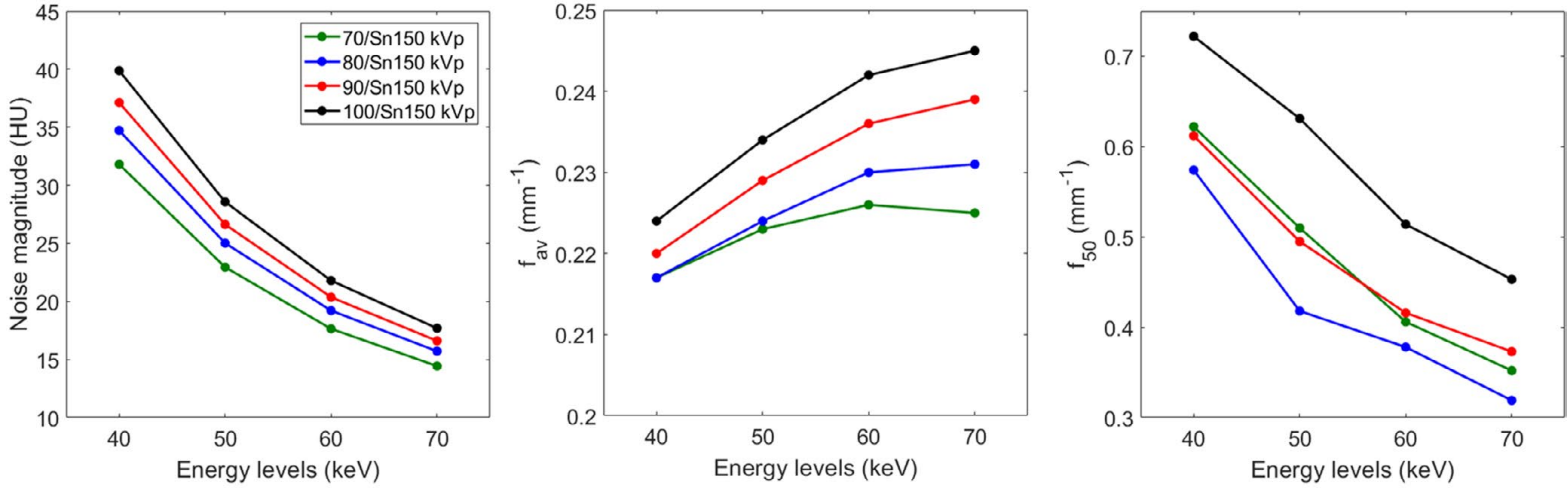
Noise magnitude, average NPS spatial frequency (f_av_), and TTF at 50% (f_50_) of acrylic insert obtained for all kVp pairs on low‐energy monochromatic images

For all kVp pairs and all acquisitions, the noise magnitude decreased by a mean of −55%± 0% between 40 keV and 70 keV (Figure 4 and Table 3). From 40 to 70 keV, the lowest values of noise magnitude were found with 70/Sn150 kVp, regardless of the energy level. Compared to 70/Sn150 kVp and for all acquisitions, noise magnitude was increased by 9% ± 0% with 80/Sn150 kVp, by 16% ± 1% with 90/Sn150 kVp and by 24% ± 1% with 100/Sn150 kVp.

**TABLE 3:** Outcomes of Noise Power Spectrum (NPS), Task‐based transfer function (TTF) at 50% (f_50_) and at 10% (f_10_) of the acrylic insert and detectability index (d’) for the two simulated lesions obtained for all kVp pairs on Virtual monoenergetic images at low‐energy levels

		Virtual monoenergetic images			
		40 keV	50 keV	60 keV	70 keV
Noise magnitude	70/Sn150 kVp	31.8 ± 0.1	22.9 ± 0	17.6 ± 0	14.4 ± 0
	80/Sn150 kVp	34.7 ± 0.2	25.0 ± 0.1	19.2 ± 0.1	15.7 ± 0.1
	90/Sn150 kVp	37.1 ± 0.1	26.6 ± 0.1	20.4 ± 0.1	16.6 ± 0.1
	100/Sn150 kVp	39.9 ± 0.1	28.6 ± 0.1	21.8 ± 0.1	17.7 ± 0.1
Average NPS spatial frequency (mm^−1^)	70/Sn150 kVp	0.22 ± 0	0.22 ± 0	0.22 ± 0	0.22 ± 0
	80/Sn150 kVp	0.22 ± 0	0.22 ± 0	0.23 ± 0	0.23 ± 0
	90/Sn150 kVp	0.23 ± 0	0.23 ± 0	0.24 ± 0	0.24 ± 0
	100/Sn150 kVp	0.23 ± 0	0.23 ± 0	0.24 ± 0	0.25 ± 0
NPS peak spatial frequency (mm^−1^)	70/Sn150 kVp	0.12 ± 0.01	0.15 ± 0	0.15 ± 0.01	0.16 ± 0.01
	80/Sn150 kVp	0.11 ± 0.01	0.14 ± 0.01	0.16 ± 0.01	0.16 ± 0
	90/Sn150 kVp	0.10 ± 0	0.13 ± 0	0.16 ± 0	0.17 ± 0.01
	100/Sn150 kVp	0.10 ± 0	0.14 ± 0.02	0.17 ± 0.01	0.18 ± 0
f_50_ (mm^−1^)	70/Sn150 kVp	0.62 ± 0.06	0.51 ± 0.04	0.41 ± 0.03	0.35 ± 0.03
	80/Sn150 kVp	0.57 ± 0.04	0.42 ± 0.03	0.38 ± 0.01	0.32 ± 0.01
	90/Sn150 kVp	0.61 ± 0.08	0.50 ± 0.03	0.42 ± 0.01	0.37 ± 0.02
	100/Sn150 kVp	0.72 ± 0.05	0.63 ± 0.02	0.51 ± 0.02	0.45 ± 0.02
f_10_ (mm^−1^)	70/Sn150 kVp	0.89 ± 0.03	0.68 ± 0.07	0.63 ± 0.04	0.61 ± 0.01
	80/Sn150 kVp	0.85 ± 0.06	0.64 ± 0.03	0.60 ± 0.03	0.59 ± 0.10
	90/Sn150 kVp	0.91 ± 0.11	0.74 ± 0.15	0.65 ± 0.04	0.63 ± 0.02
	100/Sn150 kVp	0.99 ± 0.03	0.80 ± 0.06	0.68 ± 0.03	0.64 ± 0.05
d’ values of Liver metastasis	70/Sn150 kVp	6.56 ± 0.04	6.69 ± 0.07	6.71 ± 0.10	6.67 ± 0.10
	80/Sn150 kVp	6.05 ± 0.03	6.17 ± 0.04	6.50 ± 0.02	6.28 ± 0.03
	90/Sn150 kVp	5.76 ± 0.07	5.80 ± 0.06	6.13 ± 0.05	6.03 ± 0.09
	100/Sn150 kVp	5.64 ± 0.01	5.67 ± 0.01	6.00 ± 0.02	5.90 ± 0.02
d’ values of Hepatocellular carcinoma	70/Sn150 kVp	2.34 ± 0.01	2.52 ± 0.03	2.52 ± 0.04	2.27 ± 0.03
	80/Sn150 kVp	2.15 ± 0.01	2.33 ± 0.01	2.37 ± 0.01	2.21 ± 0.01
	90/Sn150 kVp	2.02 ± 0.02	2.22 ± 0.02	2.27 ± 0.02	2.08 ± 0.03
	100/Sn150 kVp	1.97 ± 0.01	2.17 ± 0.01	2.23 ± 0.01	2.04 ± 0.01

Values are expressed as means ± standard deviations for five acquisitions performed for each kVp pair.

f_av_ and f_peak_ shifted toward higher frequencies as the energy level increased for all kVp pairs (Figure 4 and Table 3). Irrespective of the energy level, the lowest f_av_ values were found for 70/Sn150 kVp. For the same energy level, f_av_ shifted toward higher frequencies as the voltage in the tube “A” increased. The f_peak_ shifted toward higher frequencies as the tube “A” voltage increased at 60 and 70 keV. The reverse pattern was found at 40 keV and similar f_peak_ values were found at 50 keV for all kVp pairs.

#### 3.4.1 Task‐based transfer function

The f_50_ and f_10_ shifted toward lower frequencies as the energy level increased (Figure 4 and Table 3). For each energy level, the highest values of f_50_ and f_10_ occurred for 100/Sn150 kVp and the lowest for 80/Sn150 kVp. Similar values of f_50_ were found with 70/Sn150 kVp and 90/Sn150 kVp but the f_10_ values were lower with 70/Sn150 kVp and 90/Sn150 kVp.

#### 3.4.2 Detectability index

**FIGURE 5:**
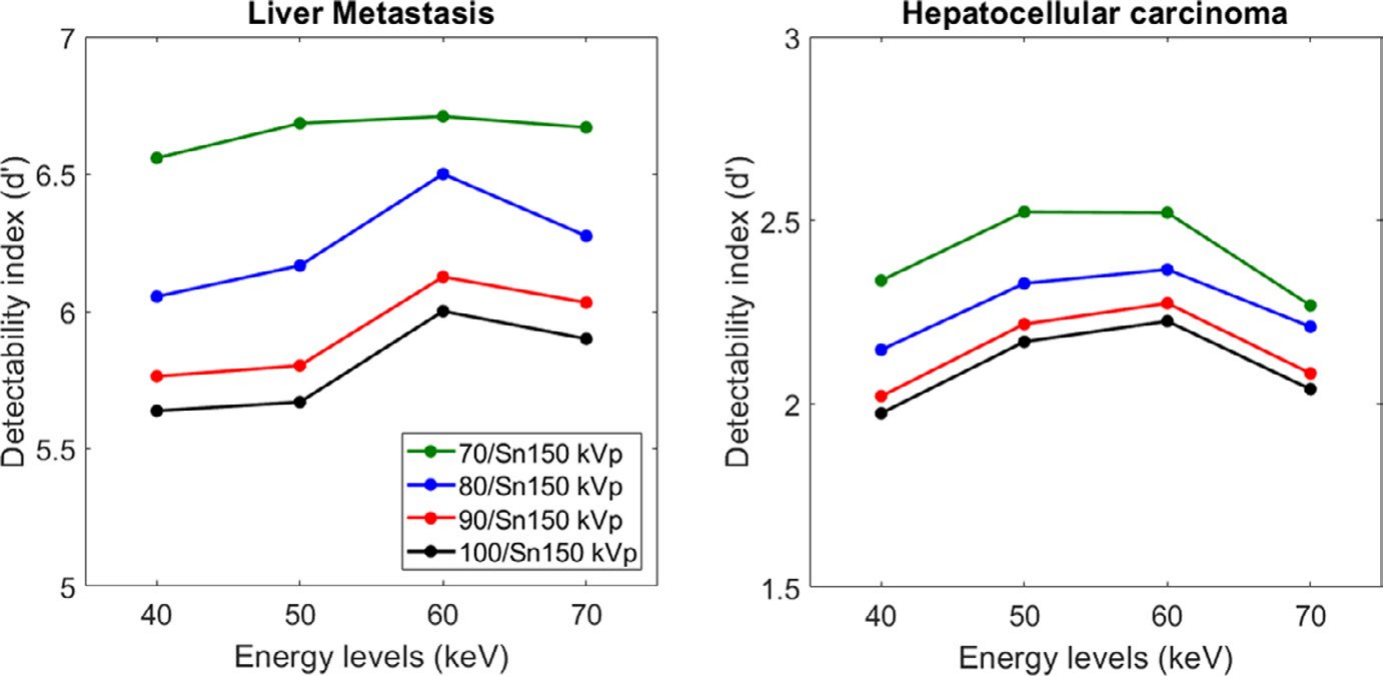
Detectability index (d′) obtained for all pairs of kVp on low‐energy monochromatic images for the liver metastasis and the hepatocellular carcinoma

Figure 5 and Table 3 depict the d′ obtained for the two simulated lesions as function of the energy level for each pair of kVp. For both lesions, the highest d’ values were found for 70/Sn150 kVp and the lowest for 100/Sn150 kVp. Compared to 70/Sn150 kVp, d’ were decreased by −6 ± 3% with 80/Sn150 kVp, by −11 ± 2% with 90/Sn150 kVp and by −13 ± 2% with 100/Sn150 kVp for all energy levels and all acquisitions.

For both lesions, d’ values peaked at 60 keV for all pairs of kVp, except with 70/Sn150 kVp. For HCC, d’ values peaked at 50 keV with 70/Sn150 kVp, but for LM, similar d’ values were found from 40 to 70 keV.

For both lesions and for each kVp pair, d’ values obtained on VMIs for all low‐keV (Table 3) were higher than to those obtained on mixed images (Table 4). The highest d’ values were obtained with 100/Sn150 kVp for both lesions.

**TABLE 4:** Detectability index (d’) obtained on mixed images generated for each pair of kVp, for the detection of two small focal liver lesions

Tube voltage (Tube A/Tube B; kVp)	Liver metastasis	Hepatocellular carcinoma
70/Sn150 kVp	4.65 ± 0.16	1.68 ± 0.06
80/Sn150 kVp	4.75 ± 0.02	1.72 ± 0.01
90/Sn150 kVp	5.12 ± 0.04	1.85 ± 0.01
100/Sn150 kVp	5.51 ± 0.08	1.99 ± 0.03

Values of the detectability index are expressed as means ± standard deviations for the five acquisitions performed for each kVp pair.

### 3.5 Iodine images

For all pairs of kVp and all iodine concentrations, the measured iodine concentrations were higher than the respective theoretical values (Table 5).

**TABLE 5:** Values of iodine measured for four iodine concentrations with the four pairs of kVp and their respective root mean square deviation (RMSD_iodine_) averaged over the four iodine concentrations and the iodine bias

		70/Sn150 kVp	80/Sn150 kVp	90/Sn150 kVp	100/Sn150 kVp
Iodine concentration (mg/ml)	2	2.4 ± 0.1	2.1 ± 0.1	2.2 ± 0.1	2.4 ± 0.2
	5	6.0 ± 0.2	5.6 ± 0.2	5.6 ± 0.2	5.2 ± 0.1
	10	11.8 ± 0.1	10.7 ± 0.1	10.6 ± 0.1	10.1 ± 0.3
	15	19.1 ± 0.7	17.3 ± 0.2	16.7 ± 0.2	15.1 ± 0.3
RMSD_iodine_ (mg/ml)		2.34 ± 0.29	1.24 ± 0.07	0.94 ± 0.07	0.29 ± 0.10
Iodine bias (mg/ml)		7.42 ± 0.51	3.78 ± 0.23	2.98 ± 0.13	0.88 ± 0.30

Values are expressed as means ± standard deviations for five acquisitions performed for each kVp pair.

For all acquisitions, the RMSD_iodine_ was the lowest for 100/Sn150 kVp (0.29 ± 0.10 mg/ml) and increased when the tube “A” voltage decreased (2.34 ± 0.29 mg/ml for 70/Sn150 kVp). Similar outcomes were found for iodine bias: 0.88 ± 0.30 mg/ml for 100/Sn150 kVp and 7.42 ± 0.51 mg/ml for 70/Sn150 kVp.

## 4 DISCUSSION

In this study, we assessed the spectral performance of the abdominal dual‐energy imaging of four X‐ray tube voltage pairs using a tin filter for the tube “B” voltage by a third generation DSCT. The noise texture, noise magnitude, and spatial resolution in mixed and separated low‐ and high‐kVp images and VMIs were evaluated. The detectability of two low‐contrast focal liver lesions was computed for VMIs and mixed images. The accuracy of iodine concentration was also measured for iodine images. We observed that, for low energy levels (ranging from 40 to 70 keV), the highest detectability and spectral performances were found using 70/Sn150 kVp. The highest detectability for mixed images and highest accuracy of iodine concentration on iodine images were for 100/Sn150 kVp.

The results of the mAs ratio found in this study were similar to those published by Krauss et al. for phantoms with diameters of 30 and 40 cm^9^. The mAs ratio is defined by the manufacturer for each pair of kVp to obtain optimal image quality. The user can change the mAs of tube “A” but the mAs of tube “B” was defined by the system. However, the choice of this ratio affects the image quality. The NPS peak outcomes showed that the mAs ratios defined for 80/Sn150 kVp and 70/Sn150 kVp gave an equivalent NPS peak for low‐kVp and high‐kVp images. Furthermore, for 90/Sn150 kVp and 100/Sn150 kVp, different NPS peak values were found between the low‐ and high‐kVp images. For 100/Sn150 kVp, the noise magnitude was 2.0 times higher in images at high‐kVp than those at low‐kVp and 1.2 lower for 90/Sn150 kVp. However, these results could also be explained by the difference in dose distribution as a function of the kVp pairs. The CTDI_vol_ results showed that the difference between the dose measured at the periphery and the center of the reference dosimetric phantom increased when the tube “A” voltage decreased. The lower this ratio, the more penetrating the photon beam and the greater the photon statistics in the images, reducing the image noise, the beam hardening, and photon starvation artifacts. The reduction of these artifacts improved the noise texture and spatial resolution of images. The outcomes of NPS spatial frequency (noise‐texture) and TTF (spatial resolution) of the acrylic insert obtained for low‐kVp images showed that the highest values were found for 100/Sn150 kVp, and these values decreased when the tube “A” voltage decreased. However, for high‐kVp images, similar values of NPS spatial frequency and TTF were found as a function of the pair of kVp because the same tube “B” voltage (Sn150 kVp) was used. Finally, the variations in the NPS peak, NPS spatial frequency, and TTF values obtained for mixed images as a function of the kVp pair were similar to those obtained for low‐kVp images. Indeed, as defined in this study, the mixed images were obtained using a DE composition factor corresponding to the low energy fraction of the image. In this study, for abdominal imaging, the weighting proposed by the manufacturer was 80% for the low‐kVp images and 20% for the high‐kVp images. This ratio was defined to obtain mixed images similar to the 120 kVp images in terms of noise and contrast enhancement. A lower DE composition value led to a decreased low energy fraction in the image and thus less contrast enhancement. Furthermore, the NPS peak was lower in the mixed images than in the low‐ and high‐kVp images. Indeed, the two sets of data used to generate these images induce a higher statistic and thus decrease the image noise. Similar variations in image noise as a function of the kVp pair were found for mixed images by Krauss et al. for 80/Sn150, 90/Sn150, and 100/Sn150 kVp.^9^ The highest NPS and TTF outcomes obtained at 100/Sn150 kVp on the mixed images leads to better detectability of the two simulated lesions. For these two lesions, detectability then decreases as the tube “A” voltage decreases.

The NPS and TTF outcomes obtained in our study on VMIs for low energy levels showed that image noise, image texture, and spatial resolution changed according to the kVp pair used. We found that the noise magnitude decreased as the tube “A” voltage decreased. These results were directly linked to the difference in spectral separation between the kVp pairs. The higher the difference between the two tube voltages (tube “A”/tube “B”), the smaller the overlap between the two‐photon spectra obtained and the better the spectral separation, resulting in less image noise. Krauss et al. demonstrated that spectral separation was higher for 80/Sn150 kVp than for 90/Sn150 kVp and 100/Sn150 kVp.^9^ We also found that the noise magnitude decreased as the energy level increased. These results are related to the decrease in the average beam energy when the keV decreases, which reduces the penetration power of the X‐ray beam and therefore increases the image noise.^33^ Furthermore, we found that average NPS spatial frequency (f_av_) shifted toward higher frequencies as the energy level increased and as the tube “A” voltage increased. The further the f_av_ shifted toward lower frequencies, the greater the image texture change (such as image smoothness). In addition, we found that TTF values shifted toward lower frequencies as the energy level increased and as the tube “A” voltage decreased, except for 80/Sn150 kVp. The further the TTF shifted toward lower frequencies, the greater was the spatial resolution reduction. These outcomes are due to the circular edge technique used to calculate the TTF by plotting the ESF. The ESF is influenced by the image noise and the contrast between the acrylic insert and the phantom background, which differ as function of keV and of kVp pairs used. Furthermore, as defined by Greffier et al., the variation of the TTF according to the energy level was related to the enhancement of the border of the acrylic insert when the keV decreased.^33^ We previously found similar f_av_ and TTF variations as function of energy levels on another DECT system.^33^

We found that 70/Sn150 kVp gave the optimal detectability of both small focal liver lesions. Indeed, d’ variations were directly related to the contrast variations of the simulated lesions and with the NPS and TTF outcomes, especially the noise magnitude variations. With a higher contrast, the detectability of LM was higher than that of HCC, whilst with the lowest noise magnitude, d’ values were highest for this kVp pair and decreased as the tube “A” voltage increased for both modeled lesions. However, the differences in d’ values between the kVp pairs were relatively modest (−13% between 100/Sn150 kVp and 70/Sn150 kVp for both lesions). In addition, we found that d’ values peaked at 50–60 keV for all pairs of kVp and were higher than mixed images obtained for each kVp pair.

We found that the accuracy of the measured iodine concentration (RMSD_iode_ and iodine bias) was higher for 100/Sn150 kVp and decreased as the tube “A” voltage decreased. The value of RMSD_iodine_ found in this study for 100/Sn150 kVp was lower than that previously reported by Sellerer et al. (0.29 vs. 0.97 mg/ml).^14^ This difference can be explained by the differences in the phantoms, reconstruction kernel, and number of concentrations of iodine used. The values of IB found in this study for 90/Sn150 kVp were higher than those previously published by Jacobsen et al.^7^ but in the same range for 100/Sn150 kVp. These variations were related to the differences between these two studies in terms of dose level and acquisition and reconstruction parameters.

Altogether, our results showed that the use of 70/Sn150 kVp is the most suitable for abdominal explorations in VMIs at low‐keV (below 70 keV). Indeed, for these energy levels, iodine contrast enhancement is preferable to improve the detection and quantification of the injected lesions, such as focal liver lesions. The results of our study show that with the 70/Sn150 kVp pair, which has the greatest spectral separation, detectability of both modeled lesions was better. However, our outcomes showed that this kVp pair generated VMIs at low‐keV that were less noisy, but more smooth and with less spatial resolution than VMIs obtained for other kVp pairs. Previous studies on iterative reconstruction algorithms have shown that a smoother image with less spatial resolution can impede the radiologist interpretation.^26^, ^27^, ^28^, ^33^ Confronted with this type of image quality, the radiologist might choose to use a higher kVp pair. In clinical practice, radiologists also use the mixed images, which approximate the conventional 120 kVp images used for almost all CT examinations. Compared to VMIs, these images are available more quickly and allow faster classical diagnosis and easier exchange with non‐radiologist physicians who are not familiar with spectral images. Lowest noise level and higher detectability were found for mixed images generated with 100/Sn150 kVp. In addition, radiologists also measure the iodine concentration from specific iodine images to determine the diagnostic thresholds in various diseases. A major error in the quantification of the iodine concentration may thus affect the detection, characterization, or monitoring of a specific lesion. We found that the accuracy of iodine concentration measured was highest with 100/Sn150 kVp. All of these outcomes imply that the choice of the kVp pair should be made according to the type of images (VMIs, iodine, or mixed images) that the radiologist will use to detect and/or characterize the lesions in practice but also the patient's morphology. Indeed, for patients’ with large morphology, the image noise will be important with increased beam hardening and photon starvation artifacts requiring using a kVp pair with a higher tube “A” voltage kVp. This was confirmed by the study carried out by Michalak et al. on phantoms of different sizes.^5^ They recommended using a kVp pair with a higher tube “A” voltage kVp with larger phantoms (or patients) to improve VMIs. The results of our study must therefore be toned down because even if we chose phantoms placed inside a body ring to represent the morphology of patients undergoing an abdomen‐pelvic CT examination, it did not take into account very large morphologies, that is, overweight or obese patients. For these patients, a pair of kVp with a higher tube “A” voltage kVp, especially 100/Sn150 kVp is highly recommended. In addition, it should be noted that Siemens defined 100/150Sn kVp as default for the dual‐energy abdominal exams for all images. Our results on phantoms now need to be confirmed in patients with different morphologies for abdominal explorations in clinical practice.

This study has several limitations. Acquisitions were performed for only one dose level and one phantom morphology; a single standard soft tissue reconstruction kernel and a single iterative reconstruction level were used. Additionally, the use of other parameter combinations may show different outcomes.^33^ Then, this study was conducted on a phantom that does not take into account the variety of the patient's body morphologies, especially overweight and obese patients. Moreover, further studies should be conducted to assess the effect of the dose level and the voxel size, the use of iterative reconstruction algorithms or other reconstruction kernels and pitch variations. Last, further studies using different sizes and types of phantoms such as anthropomorphic phantoms should be conducted to confirm our outcomes with other configurations, adding clinical relevance to our results.

## 5 CONCLUSION

In conclusion, our study assesses the noise magnitude, noise texture, spatial resolution, and detectability of two focal liver lesions for four pairs of kVp available in a DSCT for the first time. For abdominal imaging and the lowest energy levels, we found that 70/Sn150 kVp presented the lowest image noise and the highest detectability of two small focal liver lesions in VMI. However, for optimum image noise and detectability on mixed images and highest accuracy of iodine concentration measured, the 100/Sn150 kVp pair was better. The results found should now be validated in clinical practice for abdominal imaging.

## CONFLICTS OF INTEREST

The authors have no relevant conflicts of interest to disclose.

## AUTHORS CONTRIBUTION

Conception or design of the work: Joël Greffier and Djamel Dabli. Acquisition, analysis, or interpretation of data for the work: Joël Greffier and Djamel Dabli. Drafting the work: Joël Greffier and Djamel Dabli. Revising it critically for important intellectual content: Julien Frandon and Jean‐Paul Beregi. Final approval of the version to be published: All authors.

## Supporting information

Fig S1‐S3

## Data Availability

Data are available on request from the authors.
