# Supplementary material for: Impact of four kVp combinations available in a dual‐source CT on the spectral performance of abdominal imaging: A task‐based image quality assessment on phantom data
Source: J Appl Clin Med Phys. 2021 Jul 26;22(8):243–54. doi: 10.1002/acm2.13369 (PMC8364263; doi:10.1002/acm2.13369)

**Supplementary material**

**Process of Task-based transfer function**


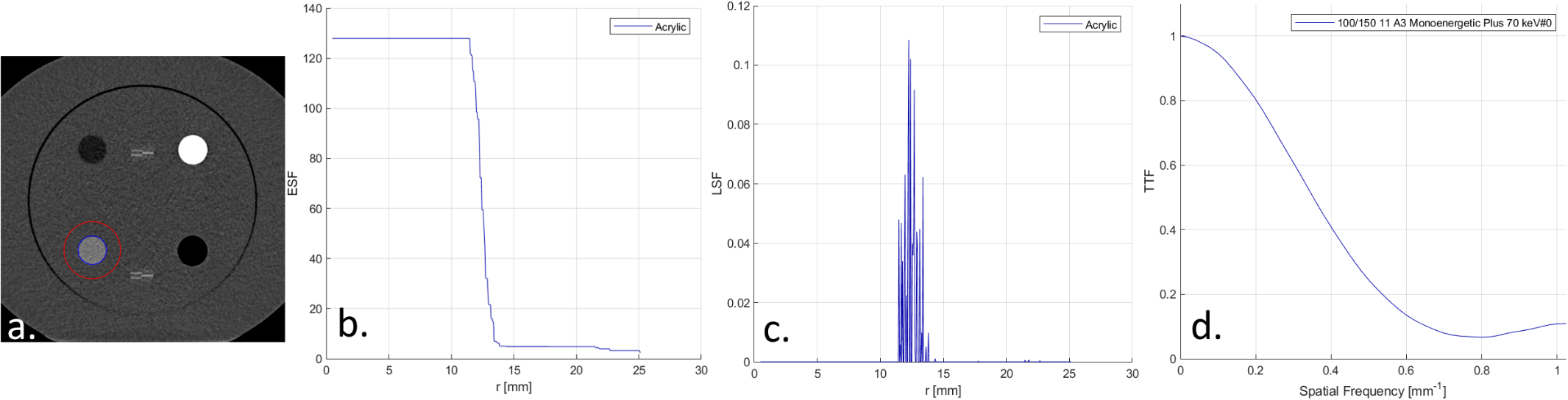


1. A ROI of 2 times the size of the insert was centered on the acrylic insert.
2. The Edge Spread Function (ESF) was plotted from the UH value of each pixel contained in the ROI brought back to their distance from the center of the insert. The resulting ESF is sampled in the form of intervals of one tenth of the pixel size and then an average over several slices of the insert is taken. This sampling is performed to improve the precision of the measurements. In addition, if the value of CNR between the insert and the solid water was lower than 15 UH, a conditioning is applied to the ESF for its monotonous curve.
3. The line spread function (LSF) was then obtained by derivation of the ESF data. The Hann filter was applied to the LSF to reduce the on the outer parts of the peak of the curve
4. TTF was computed from the normalized Fourier transformation of the LSF.

- Mean values of noise magnitude, average NPS spatial frequency (f_av_), spatial frequency of the NPS peak (f_peak_) and TTF at fifty percent (f_50_) and 10 percent (f_10_) of acrylic insert and their respective error bars obtained for all kVp pairs on low-energy monochromatic images.


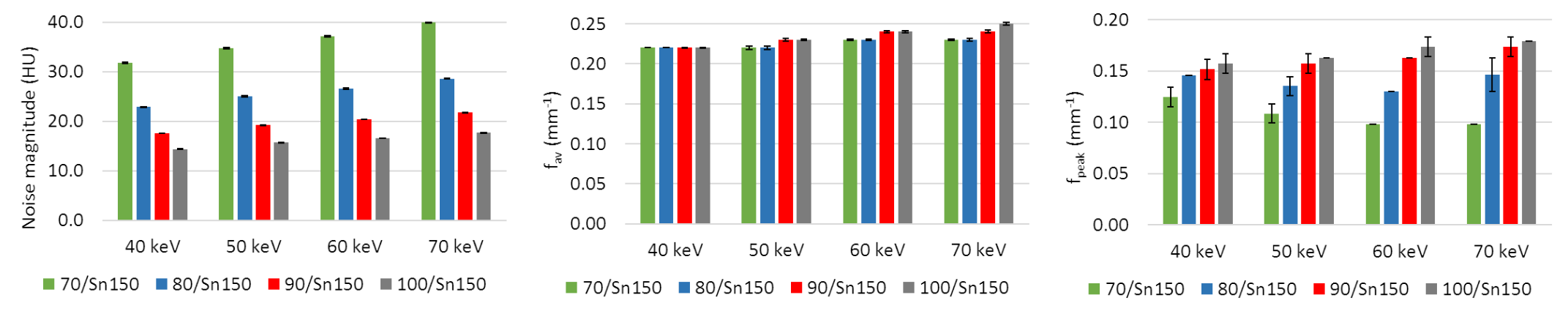


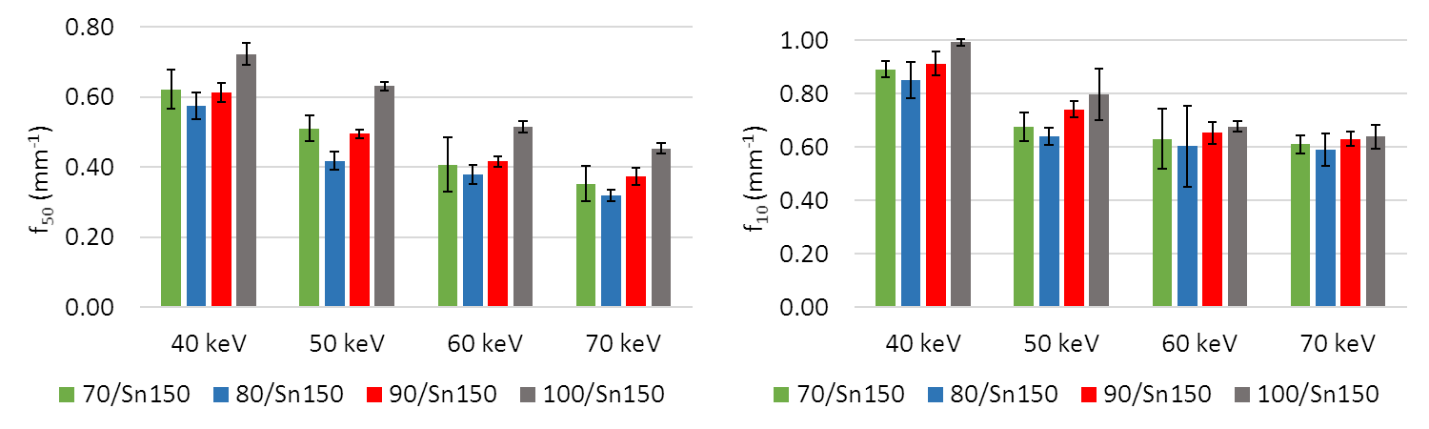


- Mean values of detectability index (d′) and their respective error bars obtained for all pairs of kVp on low-energy monochromatic images for the liver metastasis and the hepatocellular carcinoma.


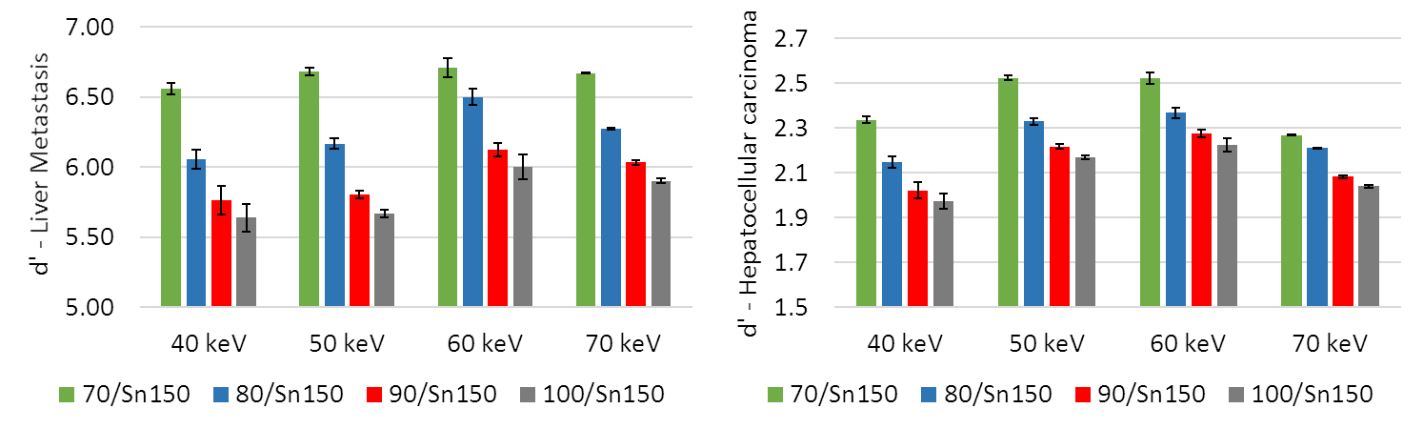

Supplement: Supplementary file 1 — Fig S1‐S3 [file ACM2-22-243-s001.docx]
